# Cytokine-mediated inflammation mediates painful neuropathy from metabolic syndrome

**DOI:** 10.1371/journal.pone.0192333

**Published:** 2018-02-06

**Authors:** Can Zhang, Joseph Ward, Jacqueline R. Dauch, Rudolph E. Tanzi, Hsinlin T. Cheng

**Affiliations:** 1 Genetics and Aging Research Unit, MassGeneral Institute for Neurodegenerative Disease, Department of Neurology, Massachusetts General Hospital, Boston, Massachusetts, United States of America; 2 Department of Neurology, University of Michigan Medical Center, Ann Arbor, Michigan, United States of America; University of Southern California, UNITED STATES

## Abstract

Painful neuropathy (PN) is a prevalent condition in patients with metabolic syndrome (MetS). However, the pathogenic mechanisms of metabolic syndrome-associated painful neuropathy (MetSPN) remain unclear. In the current study, high-fat-fed mice (HF mice) were used to study MetSPN. HF mice developed MetS phenotypes, including increased body weight, elevated plasma cholesterol levels, and insulin resistance in comparison with control-fat-fed (CF) mice. Subsequently, HF mice developed mechanical allodynia and thermal hyperalgesia in hind paws after 8 wk of diet treatment. These pain behaviors coincided with increased densities of nociceptive epidermal nerve fibers and inflammatory cells such as Langerhans cells and macrophages in hind paw skin. To study the effect of MetS on profiles of cytokine expression in HF mice, we used a multiplex cytokine assay to study the protein expression of 12 pro-inflammatory and anti-inflammatory cytokines in dorsal root ganglion and serum samples. This method detected the elevated levels of proinflammatory cytokines, including tumor necrosis factor (TNF)-α, and interleukin (IL)-6, IL-1β as well as reduced anti-inflammatory IL-10 in lumbar dorsal root ganglia (LDRG) of HF mice. Intraperitoneal administration of IL-10 reduced the upregulation of pro-inflammatory cytokines and alleviated pain behaviors in HF mice without affecting MetS phenotypes. Our findings suggested targeting HF-induced cytokine dysregulation could be an effective strategy for treating MetSPN.

## Introduction

Metabolic syndrome (MetS) is diagnosed with a constellation of central obesity, insulin resistance (IR), hyperlipidemia and hypertension [1]. It is a major contributor to increasing cardiovascular diseases and diabetes, the leading causes of morbidity and mortality in old adults [2]. In the United States, MetS affects 46.7% of individuals older than 60 years compared to that of 18.3% in the 20–39 year-old population [3, 4]. It can cause severe complications in cardiovascular, endocrine, and nervous systems. In the peripheral nervous system, MetS causes distal symmetric polyneuropathy which is presented as slowly progressive length-dependent peripheral nerve damage with predominantly sensory symptoms [5–7]. Many of these patients have painful neuropathy with neuropathic features of tingling, burning, pins and needles sensations as well as allodynia (reduced pain thresholds to nonpainful stimuli) and hyperalgesia (increased pain perception to painful stimuli) [5–7]. These painful symptoms are mostly mediated by small nerve fibers, including unmyelinated C fiber and thinly myelinated Aδ fibers in peripheral nerves [8]. Painful neuropathy from MetS (MetSPN) is often resistant to medication treatment and causes significant personal disability and societal burden [9, 10]. Early identification, prevention, and treatment of MetSPN are important parts of MetS management.

The pathomechanisms of MetSPN are still unclear. Among the 4 components of MetS, IR is the most studied for its connection to MetSPN [11, 12]. Insulin resistance of MetS includes impaired fasting glucose (IFG) and impaired glucose tolerance (IGT) which are the essential components for the diagnosis of prediabetes and diabetes. Both prediabetes and type 2 diabetes cause painful neuropathy from affecting nociceptive small fibers in a length-dependent fashion. However, recent publication demonstrates that strict glucose control does not significantly reduce the incidence or improve severity of polyneuropathy in patients with type 2 diabetes [13]. Clearly, other MetS associated mechanisms in addition to IR are involved in MetSPN [14].

Low grade inflammation is a common phenomenon associated with major MetS parameters including, prediabetes and type 2 diabetes, obesity, and hyperlipidemia [15]. This MetS-induced inflammation is associated with elevated levels of proinflammatory cytokines in tissues such as fat [16], kidney [17], skin [18], and cardiovascular systems [19–25]. The cytokine-induced inflammation is associated with increased numbers of infiltrating proinflammatory macrophages and other inflammatory cells in tissues affected by MetS complications. In addition, circulating tissue-derived factors, including tumor necrosis factor (TNF)-α, interleukin (IL)-1β, and IL-6 contribute to the development of many systemic MetS complications [26]. In parallel, reduced level of IL-10 is detected in patients with obesity, dyslipidemia, and insulin resistance [27–29]. Interleukin-10 is an anti-inflammatory cytokine that modulates the immune system via curbing the activities of pro-inflammatory cytokines [30]. Suppression of IL-10 expression can lead to development of inflammatory bowel disease and a number of autoimmune diseases [31, 32]. The significance of this dysregulation of pro-inflammatory and anti-inflammatory cytokine in the pathogenesis of MetSPN is still unclear. This lack of knowledge prevents the development of mechanism-specific therapies which potentially can target genes, proteins and/or signaling cascades underlying MetSPN.

Previously, we studied the db/db mouse, an animal model for MetSPN with type 2 diabetes. We reported increased inflammatory mechanisms mediating the development of mechanical allodynia [33]. We demonstrated that increased nerve growth factor (NGF)/p38 signaling contribute to the development of mechanical allodynia by upregulation of TNF-α, nitric oxide synthases, and cyclooxygenase 2 in the dorsal root ganglion (DRG) neurons of db/db mice [34]. In a subsequent study, we reported NGF/p38 dependent increased Tropomyosin receptor kinase (Trk) A-positive nociceptive intraepidermal nerve fiber densities (IENFDs), the activation of dermal CD68-positive dendritic cells, and CD207-positive Langerhans cell (LC) aggregations in the hind footpad skin during the period of mechanical allodynia [35, 36]. Taken together, our results suggest inflammatory mediators from DRG neurons trigger the increased numbers of nociceptive nerve fibers and inflammatory cells in the skin to mediate mechanical allodynia of MetSPN in a mouse model of type 2 diabetes.

In the current study, we use a similar strategy to study the neurogenic inflammatory mechanisms underlying MetSPN associated with prediabetes. High-fat-fed (HF) mice are widely used for studying complications from MetS associated with prediabetes [37]. The evidence of significant polyneuropathy in HF mice was previously reported [38]. In addition, similar diet treatment induces mechanical allodynia in HF mice [39]. In this study, we examine the cytokine profiles in MetSPN from high-fat-diet treatment. Using a multiarray cytokine assay, we detected a bidirectional shift of increased pro-inflammatory cytokine levels along with reduced anti-inflammatory cytokine levels in serum and LDRG of HF mice, compared to those of control-fat-diet-fed (CF) mice. We further examined the effects of exogenous IL-10 administration to reverse this cytokine dysregulation in HF mice. Our studies identify cytokine-mediated inflammation as an important contributing factor for MetSPN.

## Materials and methods

### Animals

C57Bl/6 male mice (The Jackson Laboratory, Bar Harbor, ME) at 5 wk of age were placed on either a control diet, consisting of 10% kCal fat from vegetable oil (TD.93074 from Harlan, Indianapolis, IN) or a high-fat diet, consisting of 45% kcal fat from lard (D12451i from Research Diets, New Brunswick, NJ), with 10 mice per group. Different diet groups were matched for protein and carbohydrate content. Analyses and procedures were performed in compliance with protocols established by the Animal Models of Diabetic Complications Consortium (AMDCC) (http://www.amdcc.org) and were approved by the Institutional Animal Care & Use Committee (IACUC) at Massachusetts General Hospital. All possible efforts were made to minimize animals’ suffering and the number of animals used.

### Blood chemistry

The onset of IFG was confirmed by measuring fasting blood glucose levels. After 4 hr of fasting, one drop of tail blood was analyzed using a standard glucometer (One Touch Profile, LIFESCAN, Inc., Milpitas, CA) to determine fasting glucose levels. Glucose tolerance tests were performed by measuring blood glucose at 0, 15, 30, 60, and 120 min starting a few seconds after intraperitoneal injection of 1 g/kg D-glucose. HbA1c was measured using the Helena Laboratories Test Kit Glyco-Tek Affinity Column Method (Helena Laboratories, Beaumont, TX). Fasting insulin was measured using a rat/mouse insulin ELISA Kit (Linco Research, St. Charles MO, #EZRMI-13K) according to the manufacturer's protocol. Plasma samples were run on fast-protein liquid chromatography and the fractions were assayed for cholesterol and triglycerides. These assays were performed as previously published by the Mouse Metabolic Phenotyping Center Core at the University of Washington, Seattle, Washington [40].

### Mechanical threshold measurement

The threshold for a non-noxious mechanical stimulus was assessed using von Frey filaments. The animals were placed in a Plexiglas cage with mesh flooring and allowed to acclimate for 1 h. A logarithmic series of calibrated monofilaments (von Frey hairs; Stoelting, Wood Dale, IL) with bending forces from 1 to 4 g were applied to the midplantar surface of the hind paw and pressed to the point of bending. Brisk withdrawal of the stimulated paw was recorded as a positive response. Testing began with the 1 g filament, followed by larger filaments. If no response was observed, the up–down method is used [41] with a 10 min interval to allow the animals to recover between tests. The response threshold is defined as the lightest fiber to elicit paw withdrawal. Although all responses were noted, counting of the critical 6 data points did not begin until the response threshold was first crossed. The resulting pattern of the 6 positive and negative responses was tabulated, and 50% gram threshold was calculated using the formula described previously [42]. Mechanical allodynia was determined by a significant decrease in mechanical threshold compared to the mean value of CF mice of the same age.

### Thermal threshold measurement

For plantar analgesia testing for thermal hyperalgesia, a Hargreaves’s apparatus (IITC Science, Woodland Hills, CA) was used to measure thermal thresholds as previously described [43, 44]. Mice were placed in a compartment on top of a pre-warmed glass plate to normalize the temperature of footpad skin. A visible light beam was used to stimulate the hind paws using a visible light heat source. The operator waited until the animals were at rest and then triggered a light beam. The rising temperature causes the animal to move its foot thus changing the reflected light to the paw and stopping the timer. There was a 10-min interval in between thermal stimulation to allow the animals to recover between tests Thermal thresholds were determined from the initiation of the light beam to the moments that the animal withdrawal the stimulated foot. An automatic shutoff of 10 seconds was set to prevent damage to the animals should they fail to detect the heat stimulus. No burning injury of the stimulated footpads was detected.

### Nerve conduction studies

Nerve conduction velocities (NCVs) were performed as previously described [45]. Mice were anesthetized with 30/0.75 mg/kg ketamine/acepromazine by peritoneal injection, and body temperatures were maintained at 32–34°C using a heating pad. For sural nerve NCV, recording electrodes were placed on the dorsum of the foot and stimulating electrodes on the ankle. Onset latency (ms) of the sensory nerve action potential after supramaximal antidromic stimulation of the sural nerve at the ankle was divided into the distance between the recording and stimulating electrodes (mm) to calculate the sural NCV (m/s). For sciatic-tibial motor NCV, recording electrodes were placed on the dorsum of the foot and the nerve was orthodromically stimulated first at the ankle, then at the sciatic notch. The distance between the two sites of stimulation (mm) was divided by the difference between the two onset latencies of the compound muscle action potentials (ms) to calculate the sciatic-tibial NCV (m/s).

### Immunohistochemistry

Hind footpads were collected, immersed for 6–8 hr at 4°C in Zamboni’s fixative (2% paraformaldehyde, 0.2% picric acid in 0.1 M phosphate buffer), rinsed in 30% sucrose in phosphate buffered saline (PBS) solution overnight, cryoembedded in mounting media (OCT), and sectioned at 30 μm thick before being processed for immunohistochemistry.

Tissue sections were processed for CD68, CD207, protein gene product 9.5 (PGP), and Trk A immunohistochemistry. Sections were incubated at 4° for 16–24 hr with primary antibodies: CD68 (1:200, Lifespan Biosciences, Seattle, WA), CD207 (1:1000, Abcam Biochemicals, Cambridge, MA), PGP (1:2000, AbD Serotec, Raleigh, NC), and Trk A (1:500, R & D system, Minneapolis, MN). Sections were then rinsed 3 times in PBS and incubated with secondary antiserum conjugated with different fluorophores (AlexaFluor 488, 594, or 647, Invitrogen, Carlsbad, CA) before being rinsed and mounted with ProLong^®^ Gold antifade reagent (Invitrogen). To confirm that there was no nonspecific immunoreaction, additional sections were incubated with primary or secondary antibodies alone. Fluorescent images were collected on an Olympus FluoView 500 confocal microscope using a 40 × 1.2 oil immersion objective at a resolution of 1024 × 1024 pixels. The optical section thickness was 0.5 μm. Approximately forty images per stack were flattened using the MetaMorph (Molecular Devices, Sunnyvale, CA, version 6.14) arithmetic option. Six sections were measured for each footpad. Cell density data were presented as the mean number of cells per linear mm of epidermis from a total of 12 sections per animal.

### Multi-array cytokine assay

The levels of cytokines were measured using an electrochemi-luminescence-based multi-array method through the Quickplex SQ 120 system (Meso Scale Diagnostics LLC, Rockville, MD) by previously reported methods [46–49]. In brief, the system utilizes 96-well-based high throughput readout. Specifically we utilized the murine proinflammatory panel-1 10-plex kits to detect 10 cytokines, including interferon-γ, IL-1β, IL-2, IL-4, IL-5, IL-6, keratinocyte chemoattractant */human* growth-regulated oncogene (KC/GRO), IL-10, IL-12p70, and TNF-α [50]. L4-6 DRG were collected from 8 mice per group and solubilized in T-PER (Thermo Fisher, Waltham, MA). The arrays were preincubated with 25 μl/well of assay diluent for 30 min. Following the preincubation, 25 μl of samples (from serum and DRG) or calibrator were added into the corresponding wells. The array was then incubated at room temperature with shaking for 2 h. The array was then washed three times with PBS containing 0.05% Tween 20, and thereafter 25 μl of detection antibody reagent with 2 h of incubation at room temperature. After rinsing, 2× reading buffer was added and the signals were detected by a Quickplex SQ 120 system. Cytokine concentrations in the samples were determined with Discovery Workbench (version 4) software, using the software’s curve fit model [51].

### Interleukin-10 treatment

Interleukin-10 (1 mg/kg, Sigma-Aldrich, St Louis, MO) or saline control was administered intraperitoneally starting at 13 wk of age [52]. The IL-10 treatment was repeated every other day for 3 wk. Behavior testing and data collection of body weight and fasting glucose levels were monitored weekly. Interleukin-10 treatment continued until the termination of the study at 16 wk of age. We did not detect significant side effects from IL-10 treatment.

### Real time RT-PCR

Total RNA was extracted from L4-6 DRG using the RNeasy Kit (Qiagen, Valencia, CA) according to the manufacturer’s instructions. Six DRG (bilateral L4-6) were used for each animal and a total of 4 animals were used per condition. Htc3 Reverse transcription was performed using the iScript cDNA Synthesis Kit (BioRad, Hercules, CA). Briefly, 5X iScript Reaction Mix, 1 μl iScript reverse transcriptase and total RNA template were added to a final volume of 20 μl. Reaction conditions were 5 min. at 25°C, 30 min. at 42°C and 5 min. at 85°C. PCR was performed as described previously [53] using the primer sequences: TNF-α sense 5’-AGCCGATTTGCTATCTCATACCAG, antisense 5’-CCTTCACAGAGCAATGACTCC; IL-6 sense 5’-GTCCTTCAGAGAGATACAGAAACT-3’, antisense 5’-AGCTTATCTGTTAGGAGAGCATTG-3’; IL-1β sense 5’-TCATTGTGGCTGTGGAGAAG-3’ and antisense 5’ -AGGCCACAGGTATTTTGTCG-3’; IL-10 sense 5’ -TGCTATGCTGCCTGCTCTTA-3’ and antisense 5’ -TCATTTCCGATAAGGCTTGG-3’; GAPDH sense 5’-TCCATGACAACTTTGGCATCG TGG-3’, antisense 5’-GTTGCTGTTGAAGTCACAGGAGAC-3’.

All real-time PCR reactions were carried out in 96-well PCR plates sealed with iCycler Optical Sealing Tape (BioRad). The PCR reactions contained 1X SYBR Green iCycler iQ mixture (BioRad), 0.2 μM of each forward and reverse primer, and cDNA preparation to 25 μl total volume. The PCR amplification profile was 94°C for 2 min, 35 cycles of denaturation at 94°C for 30 s, annealing at 60°C for 1 min, and extension at 72°C for 30 s, followed by 72°C for 5 min. The mRNA expression levels of the genes were tested, and amplification and fluorescence detection were performed using iCycler iQ Real-time Detection System (BioRad). At the end of the PCR, melting curves were obtained from 46 subsequent temperature increments by measuring fluorescence every 10 s with +0.5°C/step increment, beginning at 72°C. The quality of PCR products was determined by melting curve analysis. The fluorescence threshold value was calculated by the iCycler iQ system software, and the levels were normalized to values obtained for GAPDH. A non-template control [54] was run with every assay.

### Immunoblots

Following deep anesthesia, L4-6 DRG were dissected from 8 mice per condition (CF and HF mice) and homogenized in ice-cold T-PER Tissue Protein Extraction Reagent (Pierce Biotechnology, Rockford, IL) containing protease inhibitors (1 μM sodium orthovanadate and 1 μM sodium fluoride; Sigma Life Science, St. Louis, MO). Lysates were sonicated for 5 s, centrifuged and processed for protein concentration using D_C_ Protein Assay Reagents (BioRad). 50 μg of protein were boiled in 2X sample buffer, separated on a SDS-PAGE gel, and transferred to a PVDF membrane. Membranes were blocked and incubated overnight at 4°C with primary antibodies: TNF-α (1:1000, Abcam, Cambridge, MA), IL-6 (1:1000, Abcam), IL-1β (1:1000, Abcam), and Actin (1:5000, Santa Cruz Biotechnoogy, Santa Cruz, CA). After blocking with 3% BSA in TBS with 0.1% Tween 20, nitrocellulose membranes were incubated with the appropriate primary antibodies at 4°C overnight followed by secondary antibodies conjugated with horseradish peroxidase (Santa Cruz) at room temperature for 2 h. Signals were visualized using enhanced chemiluminescence reagents (ECL; Amersham Bioscience) or SuperSignal West Femto maximum sensitivity substrate (Pierce), depending on the signal strength. Images were captured using the Chemidoc XRS system and analyzed by Quantity One software (Bio-Rad). In some experiments, the nitrocellulose membranes were incubated at 60°C for 15 min in stripping solution (2% SDS, 100 mm dithiothreitol, and 100 mm Tris, pH 6.8) and then utilized for immunoblotting with an additional antibody. All experiments were repeated at least three times, and representative results are presented in the figures.

### Data presentation and statistical analyses

All data are presented as group means ± SEM. The data between CF and HF mice of the same age were analyzed using the Mann-Whitney test. Statistical comparisons between different age groups were made by one-way ANOVA followed by a post hoc Tukey’s multiple comparison test. A p-value less than 0.05 was considered statistically significant.

## Results

### HF mice develop features of increased body weight, hyperinsulinemia, and hypercholesterolemia

**Fig 1:**
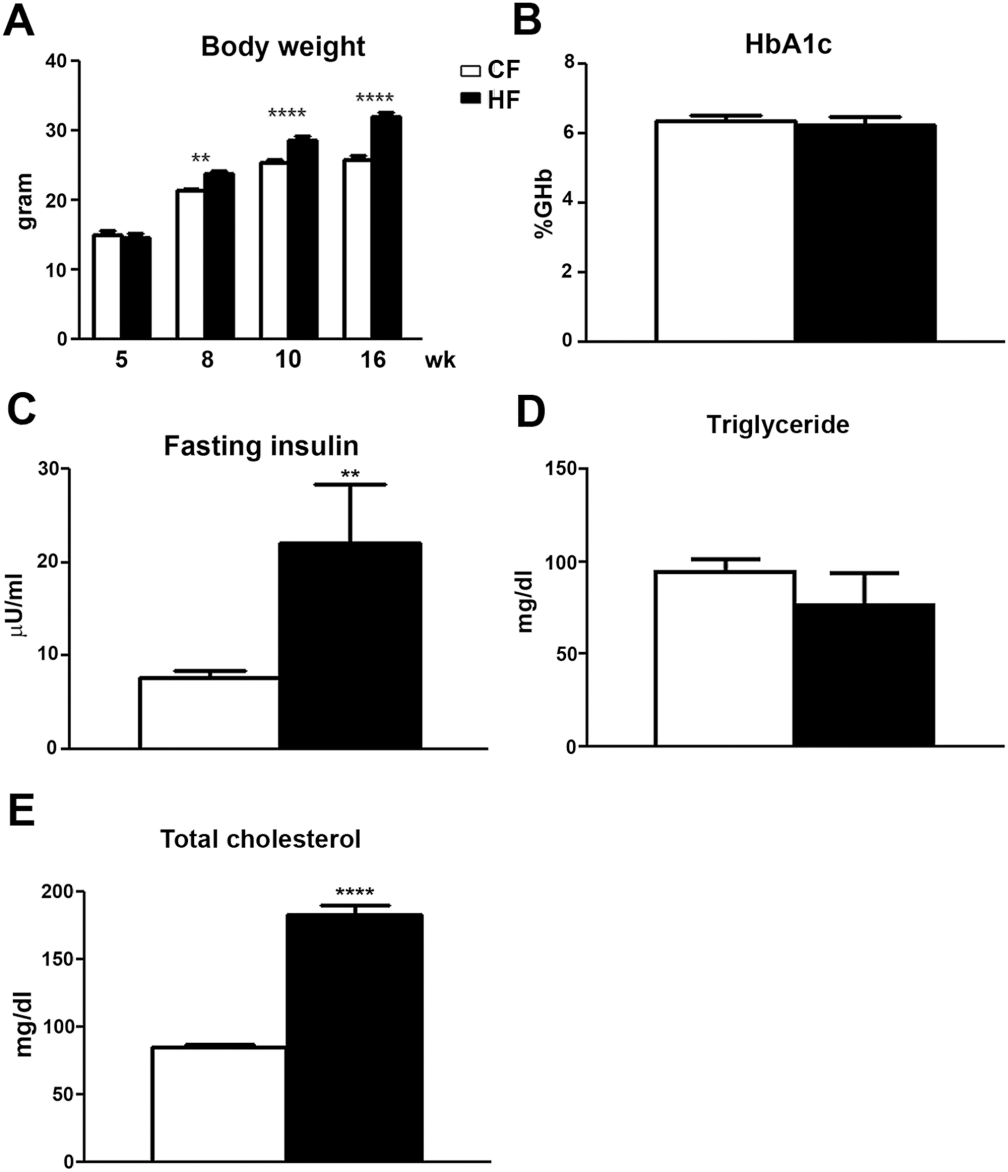
The development of MetS phenotypes in HF mice. A: Significantly increased body weight was detected starting at 8 wk of age in HF mice in comparison with CF mice; this trend continued to 16 wk of age. B: There was no change of HbA1c levels in HF mice at 16 wk of age compared to CF mice. C: Elevated fasting insulin levels were detected in HF mice at 16 wk of age. D: Increased fasting cholesterol levels were detected in HF mice at 16 wk of age. E: High-fat diet treatment did not affect the triglyceride levels at this stage. N = 10. ** p < 0.01; **** p < 0.0001, compared to CF mice of the same age.

In order to test if HF mice developed features of MetS, C57Bl/6 mice were fed either CFD or HFD starting at 5 wk of age. Body weight, fasting serum levels of HbA1c, insulin, total cholesterol, and triglyceride levels were measured in HF and CF mice from 5, 8, 10, and 16 wk of age. As demonstrated in Fig 1A, body weight of the HF mice significantly increased comparing with that of CF mice starting at 8 wk of age. This trend continued along with HFD treatment to the end of the study. The serum levels of HbA1c (Fig 1B), fasting levels of insulin (Fig 1C), total cholesterol (Fig 1D), and triglyceride (Fig 1E) were performed at 16 wk of age. The HbA1c levels of HF mice were not significantly different from those of CF mice but fasting insulin levels were significantly elevated in HF mice at 16 wk of age (Fig 1C). In parallel, fasting total cholesterol levels were significantly elevated in HF mice in comparison with CF mice (Fig 1D). In contrast, fasting triglyceride levels were not changed by HF treatment at this stage (Fig 1E).

**Fig 2:**
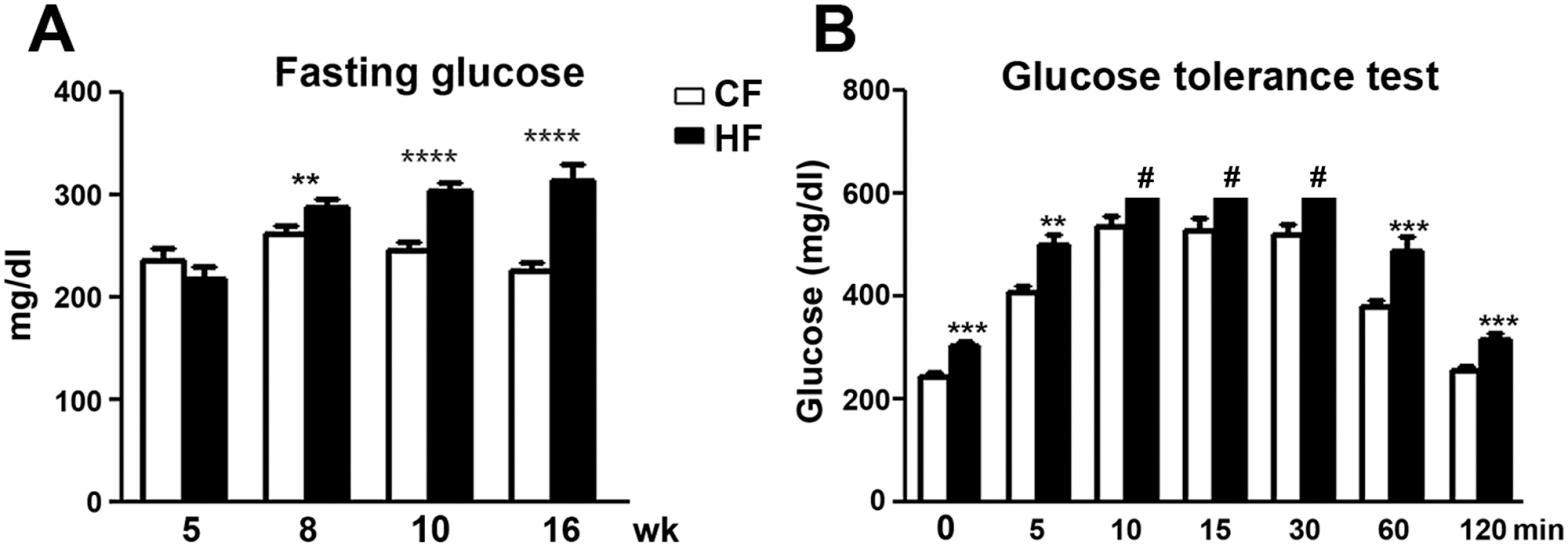
HF mice developed components of insulin resistance. A: Increased fasting glucose levels (IFG) were detected in HF mice compared to CF mice starting at 8 wk of age. This trend continued at 10 and 16 wk of age. B: G: IGT was detected in HF mice starting at 11 wk of age. In addition to the elevated fasting glucose (0 min), glucose levels were also elevated in HF mice at 5, 60, and 120 min after 1 g/kg glucose load in comparison with the levels in CF mice. N = 10. ** p < 0.01; *** p < 0.001; **** p < 0.0001, compared to CF mice of the same age. # out of range data over the limit of a glucometer.

In order to examine if HF mice developed features of insulin resistance such as IFG and IGT, we monitored fasting blood glucose levels of CF and HF mice weekly. As demonstrated in Fig 2, HF mice developed increased fasting glucose levels compared to those of CF mice starting at 8 wk of age (Fig 2A). The mean fasting glucose levels of HF mice gradually reached 1.5 folds of those of CF mice at 16 wk of age (Fig 2A). In parallel, glucose tolerance test was performed weekly after diet treatments. During the glucose tolerance test, glucose levels were obtained at 0, 5, 10, 15, 30, 60, and 120 min after 1 g/kg intraperitoneal D-glucose administration. After administration of D-glucose, elevated levels of serum glucose were detected at 5, 10, 15, 30, 60 and 120 min in both CF and HF mice in comparison with the corresponding 0 min levels (Fig 2B). Serum glucose levels from HF mice were significantly higher than those of CF mice at 0, 5, 60 and 120 min after D-glucose administration starting at 10 wk of age (Fig 2B). Glucose levels of HF mice were read as out of range (> 600 mg/dl) on a glucometer at 10, 15, and 30 min after D-glucose treatment. These results suggested that HF mice developed IGT. Impaired glucose tolerance in HF mice persisted until the end of the study (tested weekly until 16 wk of age, data not shown).

### HF mice develop mechanical allodynia and thermal hyperalgesia

**Fig 3:**
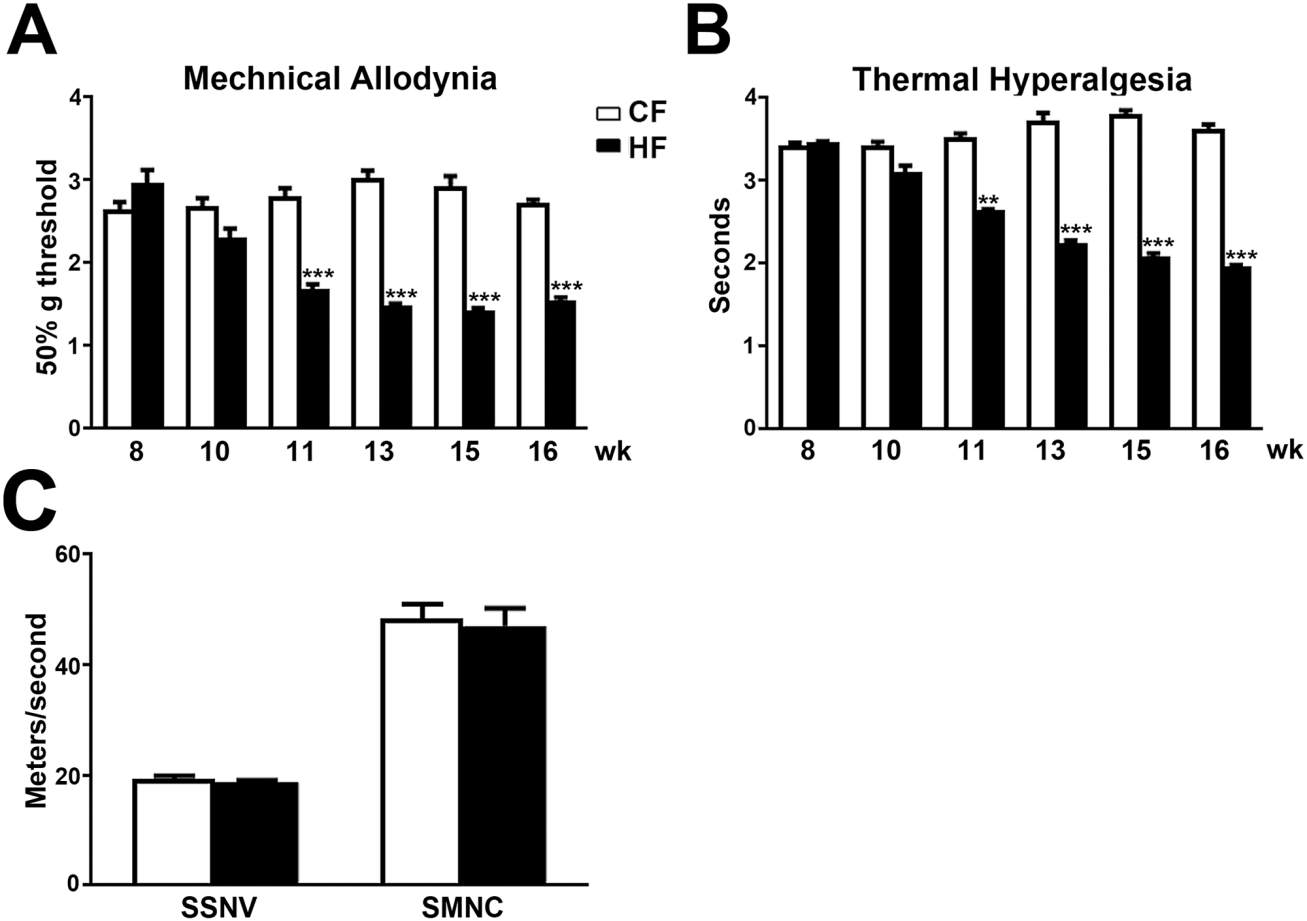
HF mice develop mechanical allodynia and thermal hyperalgesia. Reduced mechanical (A) and thermal thresholds (B) in HF mice were detected from 11–16 wk of age. C: There was no change of sural sensory and sciatic motor nerve conduction velocities at this early stage after HF treatment. *, p < 0.05;**, p < 0.01, ***, p < 0.001, compare to CF mice of the same age; Data were from 10 mice for each group.

To determine if HF mice develop pain behaviors that mimic mechanical allodynia and thermal hyperalgesia, we measured mechanical thresholds of CF and HF mice by applying Von Frey monofilaments to hind paws using the up-down protocol. Additionally, the thermal thresholds were determined by a Hargreaves’s apparatus. As demonstrated in Fig 3A, significantly reduced mechanical thresholds (defined as mechanical allodynia) were detected at 11 wk of age in HF mice in comparison with CF mice (Fig 3A). The HF-induced mechanical allodynia continued until 16 wk of age. Similarly, reduced thermal thresholds were detected starting at 11 wk of age in HF mice and persisted until 16 wk of age (Fig 3B). To determine if there is significant large fiber damage at this early stage of neuropathy, both sural sensory and sciatic motor nerve conduction studies were performed. No significant reduction of nerve conduction velocities were detected in HF mice when compared to CF mice (Fig 3C).

### Increased densities of Trk A-positive intraepidermal nerve fibers in HF mice

**Fig 4:**
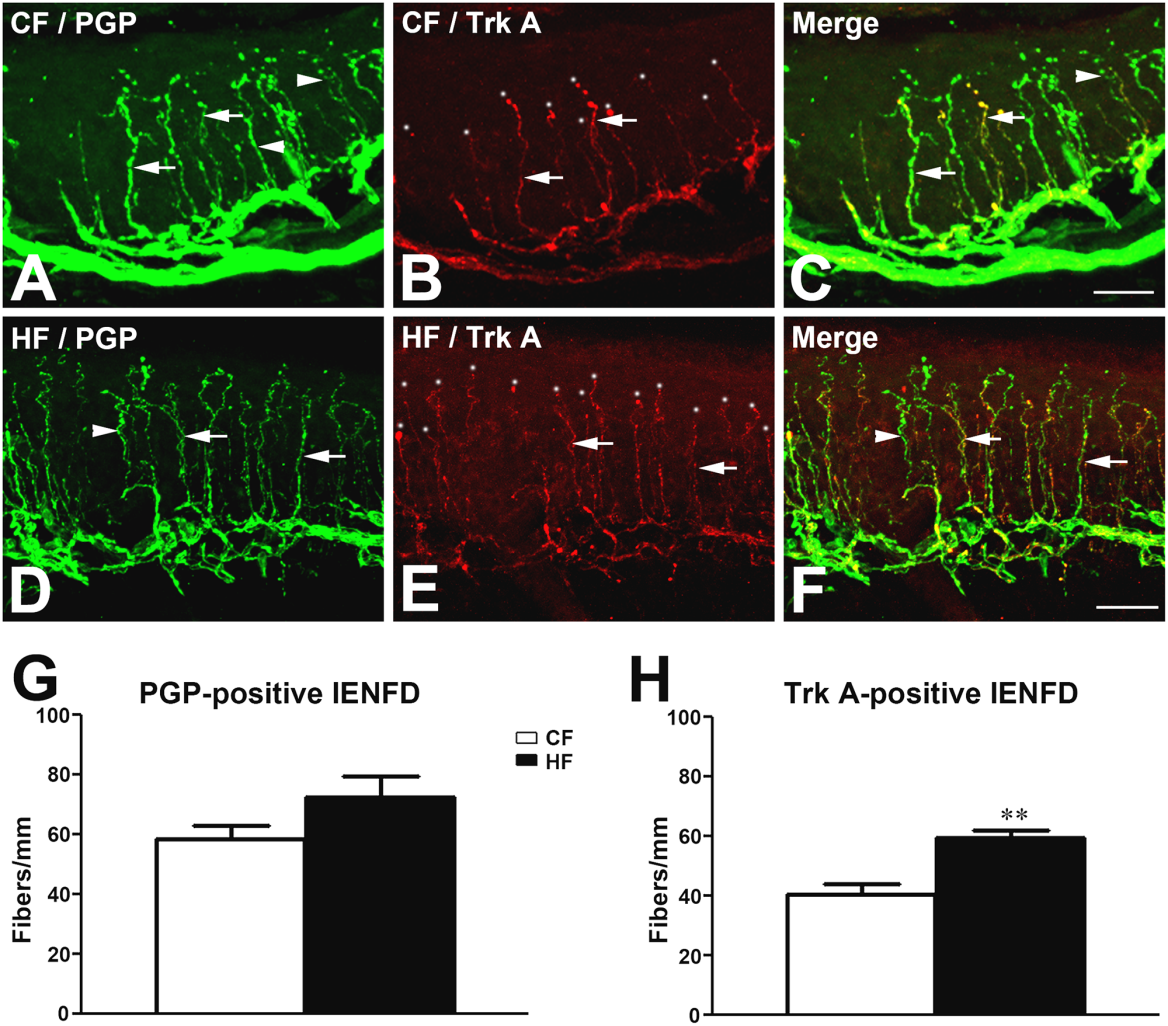
Increased Trk A-positive IENFD in HF mice. Representative confocal images of PGP (A, D) and Trk A (B, E)-positive IENFs in CF (A-C) and HF (D-F) mice at 16 wk of age. The PGP-positive IENFs included Trk A-positive (arrows) and Trk A-negative fibers (arrowheads). Quantitative studies of IENFD demonstrated increased Trk A (H) but not PGP (G)-positive IENFD in HF mice in comparison with CF mice. Bar = 50 μm. N = 10. ** p < 0.01, compare to CF mice.

Trk A is a marker for DRG neurons which innervate skin for nociception. Previously, we reported that increased Trk A-positive IENFD was associated with the development of mechanical allodynia in db/db mice [35]. To determine if this pathomechanism also mediates the pain behaviors in HF mice, immunohistochemistry of PGP 9.5 (PGP) and Trk A was performed on the hind footpad tissue sections from CF and HF mice at 16 wk of age. PGP immunohistochemistry was used to label all IENFs in the hind paw skin of CF mice (Fig 4A, arrows and arrowheads) and HF mice (Fig 4D, arrows and arrowheads). Trk A immunohistochemistry demonstrated subpopulations of PGP-positive IENFs that mediate nociception (Fig 4B and 4E, arrows and white dots). As demonstrated in Fig 4, while PGP-positive IENFD was not significantly elevated (Fig 4G), we detected significantly increased Trk A-positive IENFD in HF mice compared to CF mice (Fig 4H).

### Increased numbers of inflammatory cells in the skin of HFF mice

**Fig 5:**
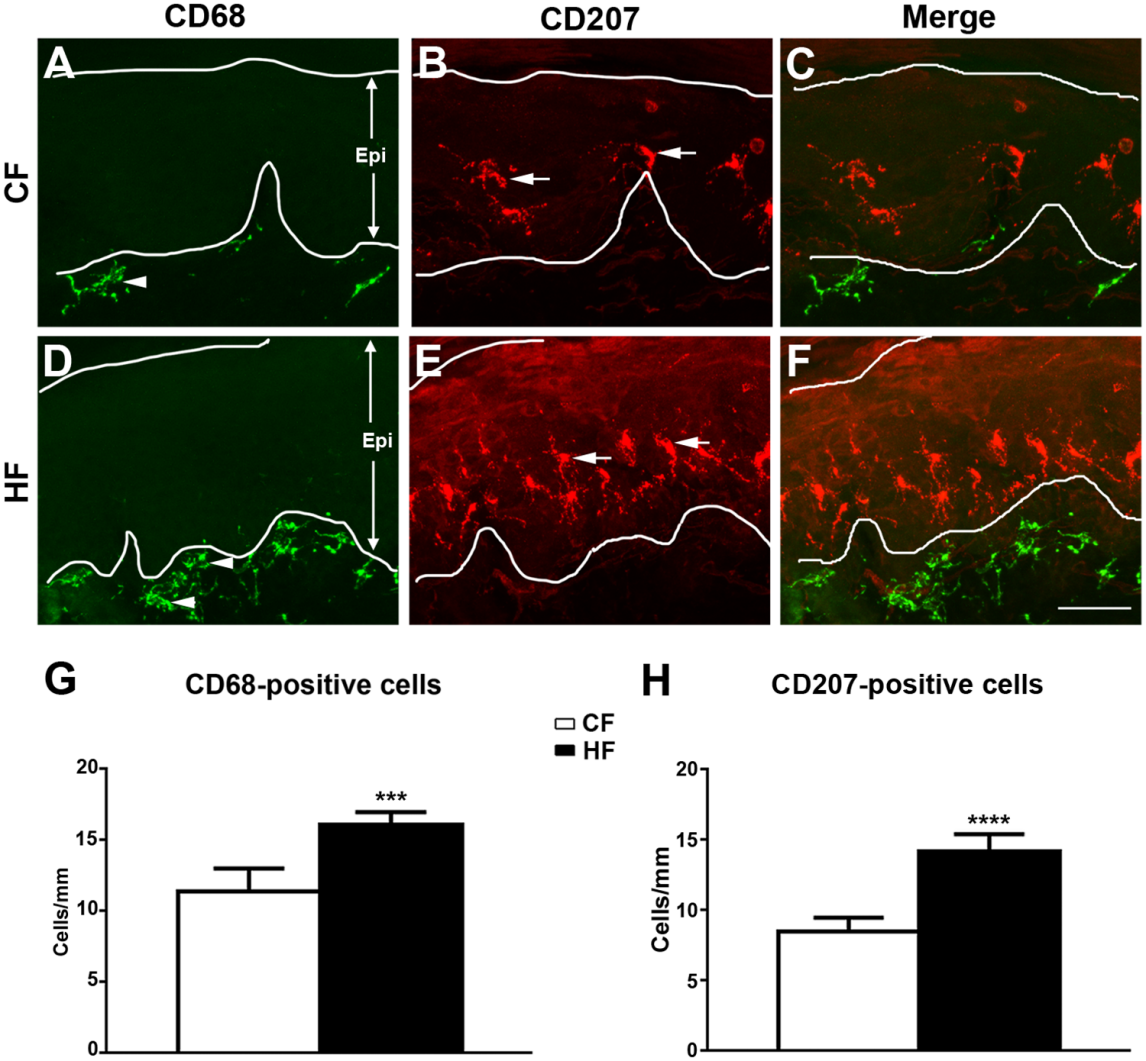
Increased CD68- and CD-207-positive cells in skin of HF mice. Representative double immunofluorescent images of CD68 (green: A,C,D,F) and CD207 (red: B,C,E,F) immunohistochemistry on hind footpads from CF (A,B,C) and HF mice (D,E,F) at 16 wk of age. Increased numbers of subepidermal CD68-positive dendritic cells (arrowheads) were detected in HF mice (compare A and D). In parallel, increased numbers of CD207-positive epidermal Langerhans cells (B, E, arrows) were detected in HF mice. Epi, epidermis. Bar = 50 μm. N = 10. *** p < 0.001; **** p < 0.0001, compared to CF mice.

We previously reported the increased aggregation of epidermal CD207-positive LCs and subepidermal CD68-positive macrophages in hind paw skin of db/db mice, a mouse model of type 2 diabetes, during mechanical allodynia [55]. Using a similar experimental approach, we performed immunohistochemistry studies for CD68 and CD207 on the hind footpads of CF and HF mice at 16 wk of age (Fig 5). Increased densities of CD68-positive cells were detected in the sub-epidermal layer of the hind footpads of HF mice compared to that of CF mice (compare Fig 5D to 5A). In a similar fashion, increased numbers of CD207-positive cells were detected in HF mice in comparison with CF mice (compare Fig 5E to 5B). Quantification analysis demonstrated significant increases of both CD68- and CD207-positive cell densities in the hind footpads of HF mice in comparison with CF mice (Fig 5G and 5H).

### Pro- and anti-inflammatory cytokines expression in LDRG after HF treatments

**Fig 6:**
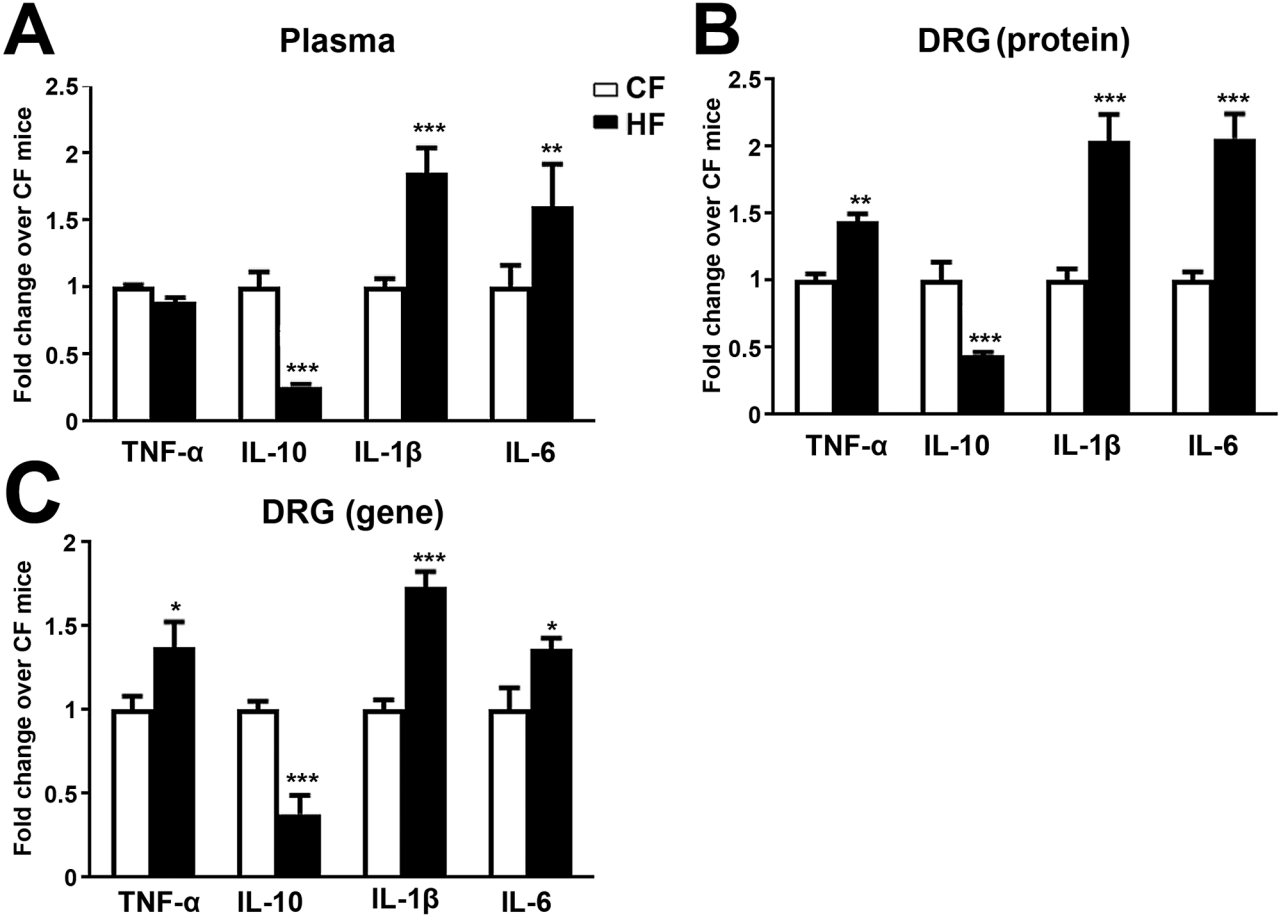
Bidirectional regulations of pro- and anti-inflammatory cytokines in serum and DRG of HF mice. A: The protein concentrations of pro-inflammatory cytokines of TNF-α, IL-1β and IL-6 as well as anti-inflammatory IL-10 were studied using serum samples by a multiarray assay system. The levels of IL-1β, IL-6 but not TNF-α were elevated in serum. In contrast, IL-10 levels were reduced in HF mice compared to CF mice. B: The protein expressions of proinflammatory cytokines of TNF-α, IL-1β and IL-6 were elevated along with reduced IL-10 expression in LDRG. C: The RT-PCR analysis detected increased gene expression of proinflammatory cytokines including TNF-α, IL-1β and IL-6 along with reduction of IL-10 gene expression in LDRG. *, p < 0.05; **, p < 0.01, ***, p < 0.001, compare to CF mice of the same age; Data were from 10 mice for each group.

We hypothesize that the dysregulation of cytokine levels can contribute to the development of pain behaviors in HF mice. We thus performed a multi-plex cytokine assay to measure the levels of 10 cytokines, including interferon-γ, IL-1β, IL-2, IL-4, IL-5, IL-6, KC/GRO, IL-10, IL-12p70, and TNF-α in LDRG and serum samples of both CF and HF mice after the development of pain behaviors at 16 wk of age [50]. As demonstrated in Fig 6, increased proinflammatory IL-1β, and IL-6 levels were detected in serum of HF mice (Fig 6A). Elevated TNF-α, IL-1β and IL-6 levels were detected in serum samples of HF mice compared to those of CF mice (Fig 6). In contrast, reduced anti-inflammatory IL-10 expression was detected in both LDRG and serum of HF mice. The levels of other cytokines in the panel were not significantly affected by HFD treatments (data not shown). Additionally, the gene expression of TNF-α, IL-1β and IL-6 was elevated in LDRG after HFD treatments (Fig 6). Our results support the presence of dysregulation of pro- and anti-inflammatory cytokines in both systemic circulation and peripheral nerves in HF mice.

### IL-10 treatment does not affect MetS phenotypes in HF mice

**Fig 7:**
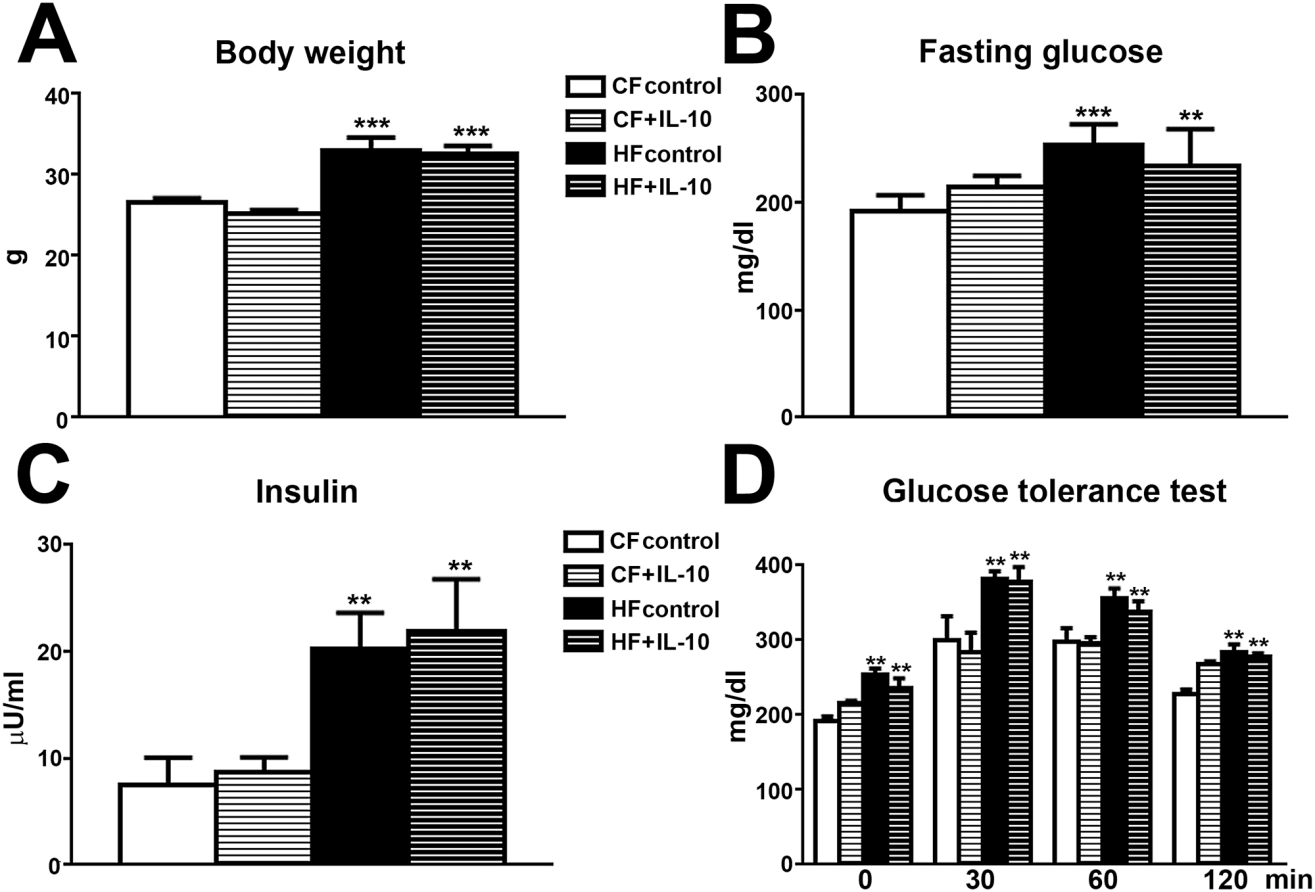
IL-10 treatment has no effect on MetS phenotypes in HF mice. IL-10 treatment did not affect high-fat diet-induced increased body weight (A), fasting glucose (B) and insulin levels (C). D: IL-10 treatment had no effect on the glucose levels at 0, 30, 60 and 120 min after intraperitoneal injection of a glucose bolus. N = 8. ** p < 0.01; *** p < 0.001, compared to CF control.

In order to test if this dysregulation of pro- and anti-inflammatory cytokines is an underlying mechanism for pain behaviors in HF mice, we administered exogenous IL-10 or control saline intraperitoneally to both CF and HF mice. The treatments were administered every other days starting at 13 wk of age for 3 wk (Fig 7). Body weight, fasting glucose and fasting insulin levels were measured at 16 wk of age. As demonstrated in Fig 7, IL-10 treatment did not affect the elevation of body weight (Fig 7A), fasting glucose (Fig 7B) and fasting insulin levels (Fig 7C) in HF mice. In addition, IL-10 treatment did not alter IGT in HF mice (Fig 7D).

### IL-10 treatment reduces mechanical allodynia and thermal hyperalgesia in HF mice

**Fig 8:**
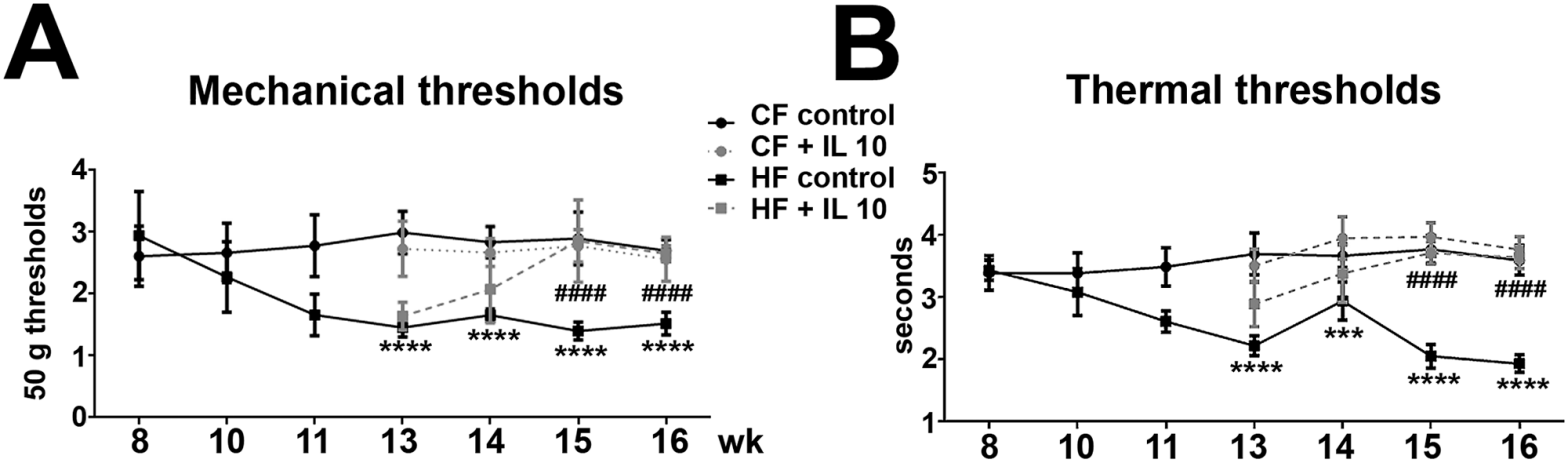
IL-10 treatment reduces mechanical allodynia and thermal hyperalgesia in HF mice. A: IL-10 treatment decreased the degrees of mechanical allodynia in HF mice. The treatment started at 13 wk of age when reduced mechanical thresholds were detected in HF mice. After 2 wk of IL-10 treatment, mechanical thresholds of HF + IL-10 group were significant elevated in comparison with HF control group and back to the CF control levels. B: In a similar fashion, IL-10 treatment reduced thermal thresholds of HF mice after 2 wk. Significantly elevated thermal thresholds of HF mice were detected at 15 wk of age compared to the levels of CF control group. N = 8. *** p < 0.001; **** p < 0.0001, compared to CF control. ## p < 0.01; #### p < 0.0001, comparing HF + IL-10 to HF control.

Both mechanical and thermal thresholds were measured weekly after IL-10 and control saline treatments in HF and CF mice. Mechanical allodynia (Fig 8A) and thermal hyperalgesia (Fig 8B) were detected in HF mice before the initiation of IL-10 treatments. Control saline treatments did not significantly change the pain thresholds in CF and HF mice. In contrast, IL-10 treatments significantly elevated the mechanical (Fig 8A) and thermal thresholds (Fig 8B) in HF mice compared to saline-treated HF mice after two weeks of treatments. By 15 wk of age, the mechanical and thermal thresholds of HF mice with IL-10 treatment were not significantly different from those of saline–treated CF mice. IL-10 treatment had no effects on the mechanical and thermal thresholds of CF mice (Fig 8A and 8B).

### IL-10 treatment reduces CD68- and CD-207-positve cell densities but not Trk A–positive IENFD in skin of HF mice

**Fig 9:**
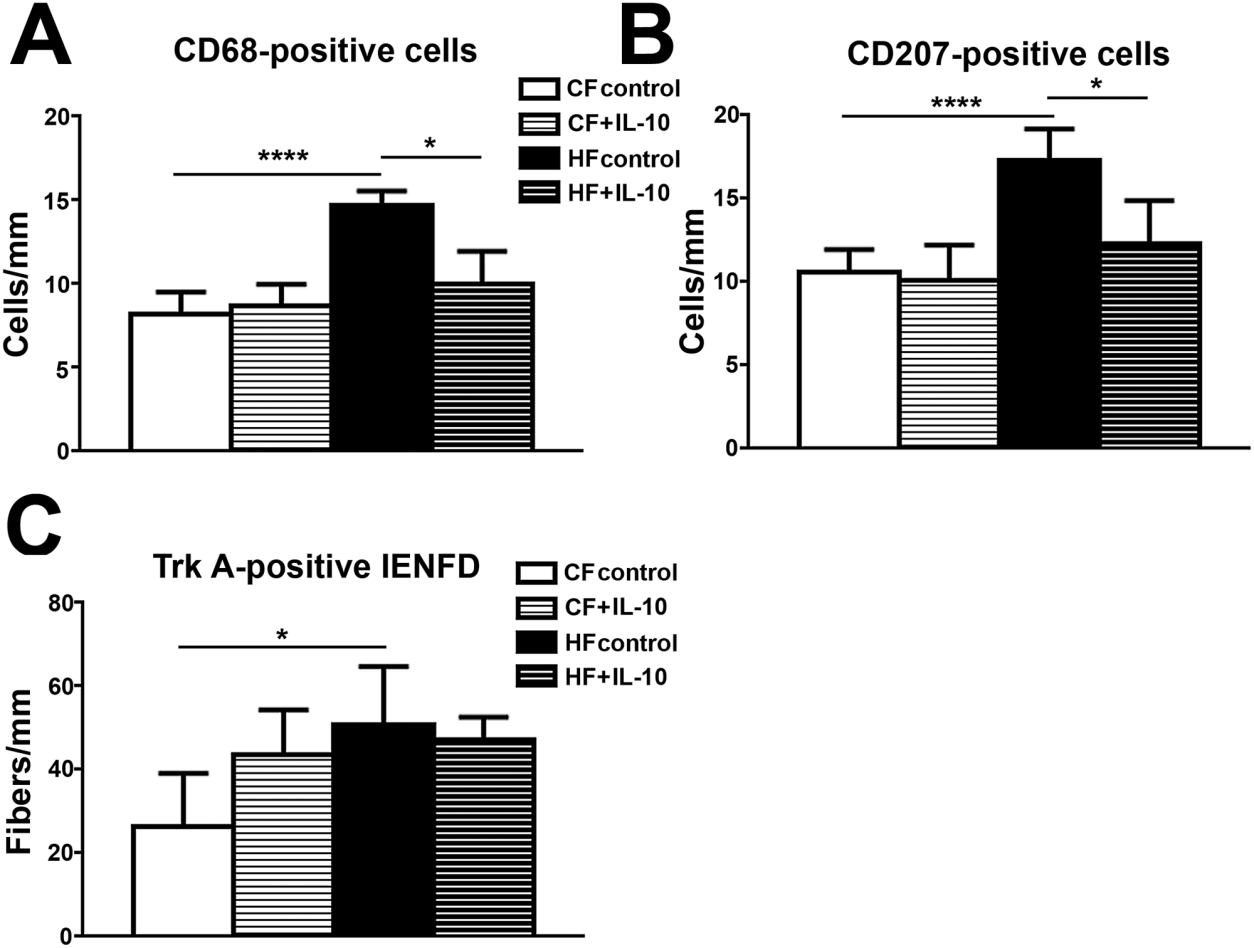
IL-10 treatment reduced CD68- and CD207-positive cells in skin of HF mice but has no effect on Trk A-positive IENFD IL-10 treatment reduced the densities of CD68- (B) and CD207-(C) positive cells in skin of HF mice. In contrast, Trk A-positive IENFD was not affected by IL-10 treatment. N = 8. *, p < 0.5; **** p < 0.0001, compared to CF control, comparing HF + IL-10 to HF control.

We next studied the effects of IL-10 treatments on phenotypes of MetSPN in HF mice. As demonstrated in Fig 8, IL-10 treatment significantly reduced the densities of CD-68- (Fig 9A) and CD-207-positive cells (Fig 9B) in skin of HF mice. The densities of both CD-68 and CD-207-positive cells in IL-10 treated HF mice were similar to those of the CF mice. However, IL-10 treatment did not affect the Trk A-positive IENFD (Fig 9C) in HF mice (Fig 9C).

### IL-10 treatment reduced TNF-α, IL-1β and IL-6 expression in LDRG of HFF mice

**Fig 10:**
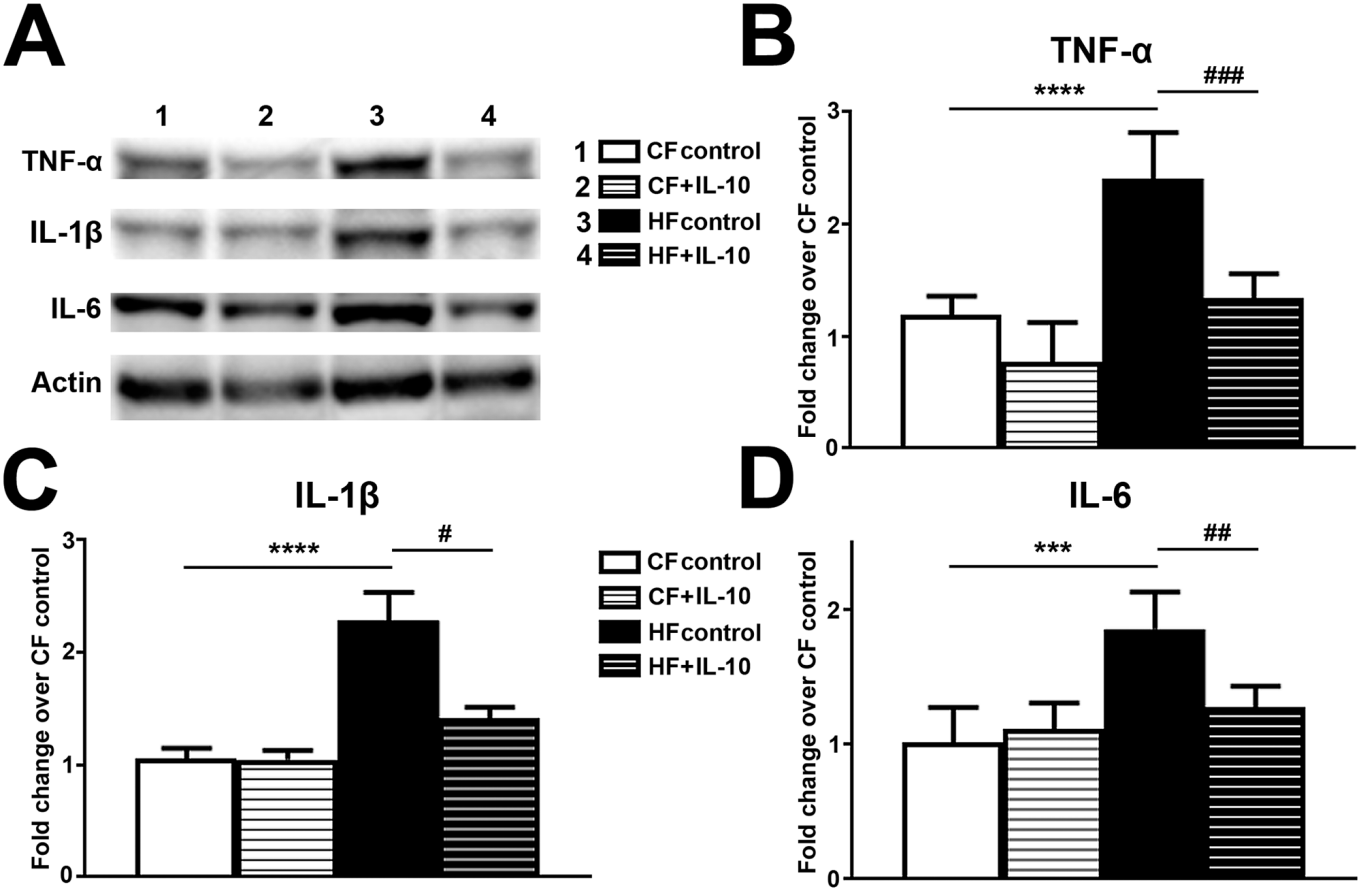
IL-10 treatment reduces protein expression of proinflammatory cytokines in DRG. The protein expression of proinflammatory cytokines including TNF-α, IL-1β and IL-6 were analyzed by immunoblots. A: Representative TNF-α, IL-1β and IL-6 immunoblots using LDRG samples of 1: CF mice treated with saline (CF control); 2: HF mice treated with saline (HF control); 3: CF mice treated with IL-10; 4: HF mice treated with IL-10, were demonstrated. Densitometric analysis of TNF-α (B), IL-1β (C) and IL-6 (D) immunoblots demonstrated IL-10 significantly reduced the expression of proinflammatory of cytokines in LDRG of HF mice. N = 8. *** p < 0.001; **** p < 0.0001, compared to CF control. ## p < 0.01; #### p < 0.0001, comparing HF + IL-10 to HF control.

To determine the effects of IL-10 administration on the levels of proinflammatory cytokines in LDRG of HF mice, we performed immunoblotting analysis of TNF-α, IL-1β and IL-6 on LDRG of CF and HF mice after control saline and IL-10 treatments (Fig 10). Immunoblots of TNF-α, IL-1β and IL-6 demonstrated upregulation of all 3 proinflammatory cytokines in LDRG of HF mice, compared to the levels of CF mice. These data confirmed the results of multiplex cytokine assay in Fig 6. In addition, IL-10 administration reduced the levels of all 3 proinflammatory cytokines in LDRG of HF mice to the levels of control-treated CF mice. In contrast, IL-10 treatments had no effect on the protein levels of TNF-α, IL-1β and IL-6 in LDRG of CF mice (Fig 10A). Densitometric analysis quantified the increased proinflammatory cytokine expression in HF mice and the significance of IL-10 reduction of TNF-α (Fig 10B), IL-1β (Fig 10C), and IL-6 (Fig 10D) in LDRG of HF mice.

## Discussion

Painful neuropathy is a common condition for patients with MetS. However, the pathomechanisms of MetSPN are not yet well understood. In the current report, we present results from studies using HF mice as an animal model of MetSPN to study the expression profiles of pro- and anti-inflammatory cytokines in HF mice in search of underlying mechanisms of MetSPN.

Our data demonstrated that HF mice develop features of MetS, including increased body weight, insulin resistance, and hypercholesterolemia, within 8 wk of HFD treatments. These constellations of symptoms mimic major parameters of MetS. In the literature, HF mice have been widely used to model MetS or its individual parameters such as obesity [56], prediabetes [38], hyperlipidemia [57] or MetS [58]. In the current study, HF mice did not have significant hypertriglyceridemia within the duration of our experiment at an early stage. Supplemental to our data, Vincent and colleagues used the same diet and reported that 30 wk of HFD treatment increases levels of triglyceride, very low density lipoprotein, and low density lipoprotein [38]. Furthermore, a similar HFD regimen with 57% kCal fat was reported to induce hypertension in rats [59]. Collectively, HFD treatment is shown to be a well established method for studying MetS.

Our study demonstrates the development of pain behaviors and elevated Trk A-positive IENFD after 8 wk of HFD treatment. Our results suggest HF mice develop MetSPN. The findings are consistent with our previous reports for painful neuropathy in a mouse model of type 2 diabetes [33, 35]. Similar to the current study, Groover and colleagues reported pain behaviors in HF mice by using a HF diet consisting of 54% kCal fat [39]. In comparison with our data, mechanical allodynia but not thermal hyperalgesia was detected within 12 wk of HF treatment. This discrepancy could be due to the use of different sources of heat stimuli. Our data of thermal thresholds are in general shorter than their results, suggesting our hear source provides stronger and faster heat stimulation which could be more sensitive for detecting thermal hyperalgesia. Increased Trk A-positive IENFD in skin of HF mice during the period of mechanical allodynia was also demonstrated in their report. This upregulation of Trk A–positive nociceptive nerve fiber during the periods of pain behaviors is consistent with our previous findings in db/db mice, suggesting it is a common mechanism among models of painful neuropathy [35, 36].

Our current findings suggest that skin inflammatory phenomena are featured mechanisms for the maintenance of mechanical allodynia and thermal hyperalgesia. These findings are similar to our previous report that demonstrated neurogenic factors such as nerve growth factor, nitric oxide, and TNF-α mediate the aggregations of CD207- and CD68-positive cells in skin during the period of mechanical allodynia in db/db mice [36]. Similar results of this inflammatory cell aggregation are also demonstrated in human studies of PN from diabetes [60] and chemotherapy [61]. These findings support that these skin inflammatory cells could be a common mechanism among PN from various etiologies.

Here, we demonstrate increased pro-inflammatory cytokines (TNF-α, IL-6 and IL-1β) and reduced anti-inflammatory cytokine (IL-10) in HF mice during the periods of pain behaviors. The dysregulation of pro- and anti-inflammatory cytokines have been widely reported in MetS studies [29]. Elevated levels of pro-inflammatory cytokines, including TNF-α and IL-6 are reported in obese individuals with MetS [26]. In addition, these proinflammatory cytokines are also considered as important mediators for the development of insulin resistance and hyperlipidemia. In addition, a plethora of published evidence demonstrates that TNF-α directly affects insulin signaling in high-fat-diet-induced obesity [26, 62]. The adipose tissue is considered as the primary source of pro-inflammatory cytokines in systemic circulation [26]. However, other tissue specific sources of proinflammatory cytokines could also mediate tissue-specific MetS complications. In parallel to our study, Cooper and colleagues demonstrated the upregulation of TNF-α, IL-1β and IL-6 in DRG of HF mice during the period of pain behaviors [63]. Our results suggest that neurogenic proinflammatory cytokines released from the epidermal small nerve fibers could be important mediators for the skin inflammatory phenomena that contribute to the development of pain behaviors in MetSPN. These findings are consistent with our previous data that demonstrated similar cell-mediated inflammation in db/db mice during the periods of mechanical allodynia [36]. This type of neurogenic inflammation has been reported in PN from other causes [64–66]. Furthermore, Moalem and colleagues provided evidence that these inflammatory reactions in the peripheral nerves could ectopically activate the nociceptive nerve fibers to induce neuropathic pain [65].

We are the very first group to report IL-10 suppression in peripheral sensory neurons of HF mice. In the central nervous system, IL-10 suppression is detected in hypothalamus after HF treatment [67]. Reduction of IL-10 level is detected in patients with obesity, dyslipidemia, and insulin resistance [27, 28]. In the current study, we demonstrate inhibitory effects of exogenous IL-10 systemic administration on pain behaviors and skin inflammatory cell aggregation without affecting IGT, IFG, hypercholesterolemia, and hyperinsulinemia. These results suggest that IL-10 suppression is a result but not the cause of MetS. Our results also support that IL-10 suppression occurs primarily in peripheral nerves but not spinal cord in HF mice. The IL-10 suppression could lead to increased proinflammatory cytokines and skin inflammatory cell aggregation. In addition to anti-inflammatory actions, IL-10 could also reduce the numbers of voltage gated sodium channels in DRG neurons to mediate analgesia [68].

Our data suggest that IL-10 improve mechanical allodynia via direct effects on DRG neurons to reduce the expression of proinflammatory cytokines [34, 68, 69]. This hypothesis is supported by evidence from previous studies which demonstrated intrathecal exogenous or viral vector-induced IL-10 over expression reducing neuropathic pain in animal model of chronic constrictive nerve injury [70]. IL-10 could reduce pain behaviors via two potential mechanisms. First, systemic IL-10 administration could reduce skin inflammation by controlling the early influx the activated dermal CD68-positive macrophages and the activation of epidermal CD207-positive LCs [71, 72]. Secondly, IL-10 treatment could also facilitate the later phase of shifting from pro-inflammatory cells to anti-inflammatory cells in peripheral nerves to reduce nerve inflammation and expedite functional recovery [73]. Further studies are needed to elucidate this matter.

In conclusion, our findings provide evidence that support the bidirectional alterations of pro- and ant-inflammatory cytokines expression in serum and LDRG in MetS. This phenomenon results in enhanced cytokine-mediated inflammation in peripheral nerves and serves as an important mechanism for the development of pain behaviors in MetSPN.

## Supporting information

S1 Fig Supporting laboratory protocols. (DOCX)
