# Supplementary material for: Cytokine-mediated inflammation mediates painful neuropathy from metabolic syndrome
Source: PLoS One. 2018 Feb 6;13(2):e0192333. doi: 10.1371/journal.pone.0192333 (PMC5800683; doi:10.1371/journal.pone.0192333)
Supplement: S1 Fig — (DOCX) [file pone.0192333.s001.docx]

Dear Editor,

Thank you for your help noticing us the requirement of data availability. Now we not only confirm our protocol and data availability statement, but also tried to address your request by uploading the laboratory protocols, and data to the suggested public repository at Figshare. Below are our laboratory protocols, and Figures (Figures 1-10) in the manuscript and their associated Digital Object Identifiers (DOIs). We believe that our current manuscript has accordingly improved and hope it can be considered for publication.

Laboratory protocol files:

<https://doi.org/10.6084/m9.figshare.>5663263

Data files:

1. [10.6084/m9.figshare.5375905](https://doi.org/10.6084/m9.figshare.5375905)
2. [10.6084/m9.figshare.5375902](https://doi.org/10.6084/m9.figshare.5375902)
3. [10.6084/m9.figshare.5375908](https://doi.org/10.6084/m9.figshare.5375908)
4. [10.6084/m9.figshare.5375935](https://doi.org/10.6084/m9.figshare.5375935)
5. [10.6084/m9.figshare.5375932](https://doi.org/10.6084/m9.figshare.5375932)
6. [10.6084/m9.figshare.5375920](https://doi.org/10.6084/m9.figshare.5375920)
7. [10.6084/m9.figshare.5375923](https://doi.org/10.6084/m9.figshare.5375923)
8. [10.6084/m9.figshare.5375929](https://doi.org/10.6084/m9.figshare.5375929)
9. [10.6084/m9.figshare.5375911](https://doi.org/10.6084/m9.figshare.5375911)
10. [10.6084/m9.figshare.5375914](https://doi.org/10.6084/m9.figshare.5375914)

Sincerely,

Hsinlin T Cheng
